# Nitric oxide in the occurrence and development of ischemic cerebrovascular disease

**DOI:** 10.4103/mgr.MEDGASRES-D-25-00117

**Published:** 2026-09-12

**Authors:** Tatiana K. Bogodvid, Dinara I. Silantyeva, Viacheslav V. Andrianov, Khalil L. Gainutdinov

**Affiliations:** 1 Zavoisky Physical-Technical Institute, FRC Kazan Scientific Center, Russian Academy of Sciences, Kazan, Russian Federation; 2 Institute of Fundamental Medicine and Biology, Kazan Federal University, Kazan, Russian Federation; 3 Volga Region State University of Physical Culture, Sport and Tourism, Kazan, Russian Federation; 4 Federal Center of Brain Research and Neurotechnologies of the Federal Medical Biological Agency of Russia, Moscow, Russian Federation

**Keywords:** dinitrosyl iron complexes, hypoxia, hypoxia-induced factor, ischemia, ischemic cerebrovascular disease, neuroprotective, neurotoxic, nitric oxide, nitric oxide synthase, nitrogen oxide depot, reactive free radical

## Abstract

Since nitric oxide is a chemically highly reactive free radical that can act as both an oxidizing agent and a reducing agent, it is assumed that it has diverse effects on biological tissues. Nitric oxide is fundamentally important for the regulation of immune, reproductive and neuromuscular system functions and is the main signaling molecule and effector that can dilate blood vessels and modulate energy metabolism. This review examines the key role of nitric oxide in the mechanisms underlying the response to hypoxia, as well as in the effects of factors induced by hypoxia. The available conflicting information allowed us to conclude that at present, there is no consensus about the role of endogenous nitric oxide in the processes occurring after ischemic cerebrovascular disease.

## Introduction

Nitric oxide (NO) is an inorganic gas, which acts as a signaling molecule in most animal and plant tissues.^1^ This small mobile reactive molecule is soluble in water and lipid membranes; thus, after synthesis, it immediately diffuses into the surrounding space. Although its lifetime in biological tissues is short, its mobility allows it to spread over significant distances between cells without difficulty. NO regulates cell metabolism and the physiological functions of the body.^2^,^3^,^4^ It is widely distributed in the nervous^5^,^6^,^7^ and cardiovascular^8^,^9^,^10^ systems. NO is fundamentally important for the regulation of immune, reproductive and neuromuscular system functions.^4^,^11^,^12^,^13^ NO also mediates smooth muscle relaxation and its regulatory roles in digestion, respiration and cardiovascular system function have been proven.^9^,^14^,^15^ Producing endogenous NO is observed in almost all the tested animal groups, as well as in plants, diatom algae, slime molds and bacteria.^1^ Investigations of the role of NO as a signaling molecule in the nervous system began shortly after its discovery.^2^,^16^,^17^ NO is known to mediate neuronal signaling in the nervous system, unlike traditional neurotransmitters. Since it is a gaseous molecule that is not stored in synaptic vesicles, NO is synthesized by neurons in response to an incoming signal and then diffuses from those neurons to neighboring cells and evokes a response.^2^,^17^ Moreover, NO is a radical that can react with various biological substrates both by nitrosylation and nitrosation. Nitrosylation occurs as the direct reaction of NO with a reagent, whereas nitrosation occurs as the indirect transfer of NO to biological nucleophiles via nitrosonium ions (NO^+^) that are previously formed as a result of NO oxidation.^18^,^19^ Additionally, one of the important functions of NO is vasodilation, the discovery of which mechanisms were awarded the Nobel Prize in this field of medicine.^2^,^20^

This review is devoted to discussing the key role of NO in the mechanisms underlying the response to hypoxia, to the analysis of sources of NO production and its depots, and to the ways and methods of its distribution in cells and tissues. The available conflicting information allowed us to conclude that, at present, there is no consensus about the role of endogenous NO in the processes occurring after ischemic cerebrovascular disease. Therefore, the main focus of our work was on the mechanisms of ischemic damage, with a focus on the features of NO production, both by enzymatic methods using different NO synthases and by nonenzymatic methods. The neurotoxic and neuroprotective functions of NO in the development of ischemia were also investigated.

## Search Strategy

A comprehensive literature search was conducted in Google Scholar, PubMed, CoLab, ResearchGate, Publons, and Springer Nature Links using the following keywords: nitric oxide (NO), ischemia, ischemic cerebrovascular disease, ischemia–reperfusion (I/R), hypoxia, hypoxia-inducible factors (HIF), nitric oxide synthase (NOS), endothelial, neuronal, inducible NOS, nitric oxide depo, intracellular receptor of NO, NO and iron complexes, reactive free radical, dinitrosyl iron complexes (DNICs), S-nitrosothiols, and neurotoxic and neuroprotective functions of nitric oxide.

## Source of Nitric Oxide

### Enzymatic synthesis of nitric oxide

The biochemical transformation of NO can be divided into three stages: generation, translocation and action.^11^ NO is enzymatically synthesized by a family of proteins with the general name of enzymes, NOS. The generation of NO is a two-stage reaction of the enzymatic oxidation of the guanidine group of L-arginine: First, the hydroxylation of L-arginine occurs with the formation of the intermediate product N-hydroxy-L-arginine, which in turn is a substrate for the enzyme NOS.^21^,^22^ Second, the N-hydroxy-L-arginine molecule is oxidized to L-citrulline with the formation of NO.^23^ The presence of the enzyme NOS is necessary for NO biosynthesis. There are three isoforms of NOSs: endothelial, neuronal, and inducible. All three isoforms have homologous regions in the oxygenase and reductase domain but exhibit different properties, reflecting their specific functions in living organisms. The homologous regions of NOS consist of the heme domain associated with flavin mononucleotide/flavin adenine dinucleotide reductase, which transfers electrons from nicotinamide adenine dinucleotide phosphate to heme.^24^,^25^ The reaction of hydroxylation occurs with the participation of the cofactor tetrahydrobiopterin (BH4), which is calcium-calmodulin- and heme-dependent and is inhibited by carbon monoxide. In the translocation stage of the reaction, L-citrulline and free NO radicals are formed. It is assumed that the hydroxylated form of L-arginine is converted to L-citrulline with the simultaneous formation of NO.^4^,^26^,^27^

Endothelial NOS (eNOS) and neuronal NOS (nNOS) are active in the presence of Ca2+ ions, whereas the activation of inducible NOS (iNOS) occurs only after induction by cytokines or other inflammatory factors.^28^,^29^ This selective dependence on Ca^2+^ ions is responsible for binding eNOS and nNOS to the Ca^2+^-calmodulin complex, which is necessary for their activation. iNOS cannot bind irreversibly to the Ca^2+^-calmodulin complex.^21^,^28^ It is known that the calmodulin binding site of eNOS is directly involved in the association of the enzyme with the cell membrane, and this association can prevent the reversible binding of eNOS with the Ca^2+^-calmodulin complex before the activation of eNOS.^23^,^30^ The catalytic activity of nNOS requires interaction between the two oxygenase domains of nNOS dimers, as well as the formation of a link between the oxygenase and reductase domains via calmodulin.^31^ Thus, the demerization of nNOS is necessary for catalyzing the conversion of L-arginine to L-citrulline with the release of NO. The monomeric subunit of nNOS can function independently and catalyze reactions involving the formation of superoxide anions and peroxynitrite.^23^,^32^ This property of the nNOS monomer is dependent not only on the disruption of dimerization but also on conditions of a deficiency or absence of L-arginine, tetrahydrobiopterin (BH4) and heme. In this case, an electron is transferred from nicotinamide adenine dinucleotide phosphate to flavins, resulting in the formation of oxygen superoxide.^29^

The unique properties of each of the three NOS isoforms are related to the localization of their production. Thus, eNOS is mainly expressed in the cardiovascular system; its main source is vascular endothelial cells, and eNOS can also be expressed in cardiomyocytes, platelets and hippocampal neurons.^23^,^33^ The main feature of eNOS produced in vascular endothelial cells is its ability to be activated not only by neurohumoral factors but also by hemodynamic parameters, such as the velocity of blood flow.^23^,^34^ Thus, according to the literature, eNOS can have a dual effect: in large vessels, it serves mainly to produce NO, which has a vasodilating effect through the activation of cyclic guanidine monophosphate, whereas in the resistive vessels of the microcirculatory bed, eNOS activates the production of superoxide, which causes vasodilation through the endothelium-dependent hyperpolarization system.^35^ In addition, eNOS induction can be caused by the presence of adrenaline, prostaglandin E and bradykinin.^35^,^36^ eNOS is maintained in an inactive state by binding to the protein caveolin, which is present in the caveolae of the vascular epithelium and is an important step in the activation of the enzyme by intracellular signaling cascades.^33^

nNOS is the largest molecule of all three types of NOS, as it has an additional chain formed from 300 amino acids, which contains a domain that is responsible for the association of the enzyme with the cell membrane. This association regulates the intracellular localization of nNOS and sensitivity to calcium-dependent activation in some neurons.^23^,^37^ In the synapses of brain neurons, the biosynthesis of NO is mediated by the calcium current activation of nNOS, which occurs through N-methyl-D-aspartate (NMDA) receptors.^38^ nNOS in these neurons contains a domain homologous to a similar domain of the protein in the postsynaptic membrane associated with the cytosolic tail of the NMDA receptor. Thus, activation of the NMDA receptor leads to an increase in nNOS activity and an increase in NO production, which is involved in the processes of neuronal plasticity.^38^,^39^ The complex of nNOS with the cell membrane, through a chain of cytoplasmic reactions involving the capon protein, may regulate transcription in the neuronal nucleus.^39^

Neurons expressing nNOS are found in many brain structures, but most abundant populations are in the hypothalamus and thalamus, as well as in some brain structures involved in processing sensory information, particularly in the olfactory bulbs or in the inferior colliculus of the midbrain. Whereas, the most specific neurons are found in the hippocampus. Fewer populations of neurons expressing nNOS are found in structures involved in motor control, such as the striatum and substantia nigra.^17^ An alternative isoform of nNOS is found in fast-type skeletal muscle cells. In these cells, nNOS is linked to the dystrophin-associated protein complex.^40^ When calcium concentrations increase due to muscle contraction, the nNOS isoform catalyzes the formation of NO, which diffuses out of the muscle fibers and participates in the dilation of local blood vessels.^41^ A similar nNOS isoform is present in myocardial cells and is located on the sarcoplasmic reticulum of cardiomyocytes, where it regulates the reuptake of Ca^2+^ ions from the intracellular space through the Ca^2+^ pump and is possibly involved in the activation of ryanodine receptors.^42^,^43^

Although eNOS is present in vascular smooth muscle cells, nNOS is also involved in the regulation of vascular function. It has been shown that nNOS performs an important function in the regulation of cerebral circulation, participates in the autoregulation of renal blood flow by regulating the lumen of the afferent arteriole of the renal capsule and participates in the vasodilation of coronary arteries.^43^,^44^,^45^

Under normal physiological conditions, iNOS is rare, since the expression of this enzyme is induced by inflammatory mediators and is independent of Ca^2+^.^23^ iNOS is primarily activated in immune cells of the blood and tissue macrophages by interferon or interleukin-1.^29^,^30^,^46^ Some investigations have shown that iNOS can be expressed in endothelial cells, cardiomyocytes, and smooth muscle cells under the influence of inflammatory mediators or endotoxins.^23^,^46^ As the activation of iNOS does not require an increase in the concentration of Ca^2+^, it can produce greater quantities of NO than other synthases.^25^ In the healthy brain, iNOS can be expressed in limited amounts in the hypothalamus, thalamus, hippocampus and amygdala during postnatal development, whereas its activation by the inflammatory process occurs only in microglia.^47^

### Non-enzymatic synthesis of nitric oxide

In addition to the biochemical synthesis of NO with the help of enzymes, there are also the production of other non-enzymatic NO. The non-enzymatic pathway is usually understood as the reduction of nitrites or nitrates to NO. This reaction occurs either as a disproportionation of nitrite or nitrous acid during acidification of the medium or as a direct reduction of nitrite (for example, with Fe ions).^48^ Nitrite reductase systems provide a sufficiently large amount of NO in the body. These include nitrite reductase components associated with hem-containing proteins.^8^,^27^,^48^ On the one hand, nitrites are endogenously produced as oxidative metabolites of NO, but they also function as NO donors that are activated by several cellular proteins under hypoxic conditions.^49^,^50^

## Nitric Oxide Depots

Synthesized NO is able to rapidly diffuse into tissues and interact with various biological targets involved in a range of physiological processes. After it interacts with biological targets, NO is inactivated. There are two main mechanisms that stabilize the unpaired electron of the NO radical: reaction with other free radicals and interactions with the d-orbitals of transition metals.^7^,^26^ Estimation of NO synthesis and dissociation revealed that physiologically relevant NO concentrations in the range of 100 μM to 1 nM varied within very small limits upon direct oxidation in solution and could be maintained for long periods of time.^50^ Thus, these findings demonstrated that intra- and extracellular processes in living systems developed against the background of a relatively constant level of NO and could decrease or increase the NO content under physiological conditions.

In addition, many biological compounds in the body serve as NO depots.^11^,^51^ The main factors involved in NO deposition and transport are S-nitrosothiols and DNICs.^3^,^48^,^52^ NO can interact with iron complexes in three ways: (a) binding to iron, (b) reacting with divalent iron complexes and (c) reacting with trivalent oxygen-containing complexes.^7^,^53^ Copper and iron are critical cations for maintaining homeostasis due to their catalytic role in physiological processes.^54^ Professor A. F. Vanin reported that DNICs with thiol-containing ligands, which appear in animal tissues in paramagnetic mononuclear and diamagnetic binuclear forms after NO synthesis, provide the stabilization and deposition of NO (in the form of protein-bound DNICs) and its transfer (in the form of low-molecular dinitrosyl complexes) to targets of its biological action, acting not only as NO donors but also as donors of NO^+^.^55^

## Pathways of Nitric Oxide Distribution and Its Binding Sites

NO is a molecule that is diffuse.^2^ Molecules of NO can easily penetrate through membranes and bind with its biological receptor in soluble guanylyl cycles.^4^,^20^,^29^ A continuous supply of oxygen is necessary for the survival of living organisms. Understanding the regulation of oxygen supply in the circulatory system evolved from the concept of passive oxygen transfer by red blood cells to the recognition of the complex interactions among hemoglobin, oxygen, carbon dioxide, and NO. The three-gas respiratory cycle ensures sufficient oxygen and nutrient delivery for the metabolic needs of biological tissues.^56^

The dominant targets of the biological action of NO are heme-containing proteins. The biochemical activity of these proteins depends on the interaction of NO with iron from their hem-subunit.^48^,^55^,^57^ DNICs are relatively stable forms of NO in cells. The formation of these complexes involves thiol groups of proteins or thiols with low molecular weights (for example, cysteine or glutathione (GSH)) and non-heme iron.^51^,^52^ Thus, dinitrosyl iron–sulfur complexes serve as among the most important depots of NO and transport this molecule to binding sites.^55^,^58^ Electron paramagnetic resonance (EPR)-detectable mononitrosyl iron complexes with diethyldithiocarbamate were experimentally shown to form both in the absence and in the presence of dithionite as a result of the reaction of NO from nitrite with Fe^2+^-diethyldithiocarbamate and Fe^3+^-diethyldithiocarbamate complexes. These complexes could be present in these tissues predominantly in their diamagnetic, EPR-undetectable binuclear form.^59^

The activity of NO is mediated by its binding with soluble guanylate cyclase, its biological receptor.^17^,^19^ This stimulates the production of cyclic guanosine monophosphate (cGMP) from guanosine triphosphate. The secondary messenger cGMP is a powerful regulator of cellular metabolism and affects cell functions. Once synthesized, the NO molecule quickly diffuses or is transported to tissues, which requires the effects of NO. Then, it binds with guanylate cyclase and stimulates the production of cGMP. cGMP subsequently activates protein kinase G and cyclic nucleotide-gated ion channels, which regulate smooth muscle tone and neurotransmission.^27^,^60^,^61^ Given that the lifetime of NO molecules is short, the transport system is of great importance for mediating their effects. Blockade of the soluble guanylyl cycle with a pharmacological inhibitor abolished NO donor-induced angiogenesis.^11^,^62^,^63^ Thus, the soluble guanylyl cycle is a main intracellular receptor of NO.

## Physiological Roles of Nitric Oxide

### Hypoxia and nitric oxide

Hypoxia is a pathological process that occurs during biological oxidation under conditions of oxygen deficiency; it is important in the pathogenesis of many diseases.^64^,^65^,^66^ When the oxygen content in the inhaled air decreases or cerebral blood flow is disrupted, the oxygen supply decreases in the brain, and cerebral ischemia occurs. In turn, ischemia can result in ischemic stroke, accompanied by brain tissue damage and brain dysfunction.^67^,^68^ The main factors associated with ischemia are acute arterial thrombus formation, chronic stenosis of the main artery and spasm of arterial vessels, which can lead to local or generalized oxygen deficiency.^13^,^59^,^69^ In this regard, the study of stroke pathogenesis and methods for correcting impaired brain function seems important from both theoretical and practical perspectives.^70^,^71^,^72^,^73^

The function of neurotransmitter systems, including the NO system, is disrupted during hypoxia and cerebral ischemia. Since NO is a chemically highly reactive free radical capable of acting as both an oxidizing agent and a reducing agent, it can have diverse effects on biological tissues.^51^,^62^ NO is the main signaling and effector molecule that mediates the response to hypoxia, due to its unique ability to dilate blood vessels (improving blood flow and tissue oxygen supply) and modulate energy metabolism (reducing oxygen consumption and stimulating the use of alternative metabolic pathways).^74^ However, the knowledge concerning the functional role of endogenous NO in the process of developing neuronal injury is contradictory and confusing.^75^,^76^,^77^,^78^,^79^

NO and reactive nitrogen species are important signaling molecules that are involved in several different biological pathways and pathologies, including systemic blood pressure, regulation of hypoxic genes (hypoxic signaling), regulation of stress response pathways, host–microbe interactions, immune signaling, and apoptosis. Importantly, cells have two different pathways for NO synthesis, and these pathways may function differently in the HIF signaling system. The first pathway is L-arginine-dependent NO synthesis by NOS, which has been described previously. However, there is also a second pathway: nitrite-dependent synthesis of NO by mitochondrial cytochrome C-oxidase. It involves the reduction of nitrite to NO and is catalyzed by several heme-containing proteins. Currently, the NOS-independent pathway of biological NO synthesis is considered a circulatory reservoir of NO in mammals. These two pathways of NO synthesis are regulated by different oxygen levels (**Figure 1**). While the arginine-dependent pathway is optimal at high oxygen levels and is oxidative, the NO-nitrite-dependent pathway occurs under low oxygen conditions and is reductive.^80^ Thus, tissue oxygen concentration may regulate which pathway and under what conditions is active.

**Figure 1:**
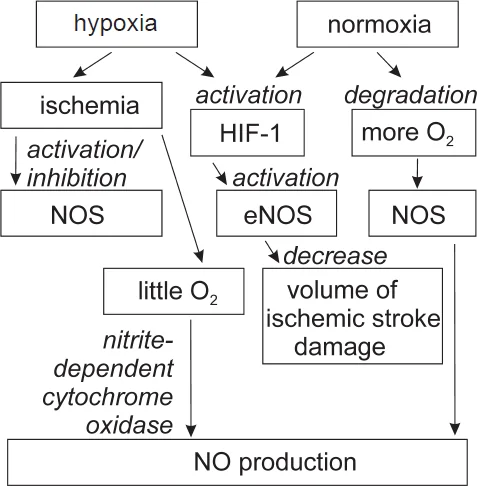
NO production after ischemia at high and low levels of O_2_. The first pathway is L-arginine-dependent NO synthesis by NOS. However, the L-arginine pathway of NO synthesis becomes less efficient when oxygen levels decrease and probably does not exist under hypoxic conditions. However, there is also a second pathway: nitrite-dependent synthesis of NO by mitochondrial cytochrome C-oxidase. Currently, the NOS-independent pathway of biological NO synthesis is considered a circulatory reservoir of NO in mammals. Created with CorelDRAW 2021. eNOS: Endothelial nitric oxide synthase; HIF-1: hypoxia-inducible factor; NO: nitric oxide; NOS: nitric oxide synthase; O_2_: oxygen.

### Hypoxia-inducible factor and nitric oxide

Adaptation to low oxygen tension (hypoxia) in cells and tissues develops as a result of the induction of the transcription of a number of genes that are involved in angiogenesis, iron metabolism, glucose metabolism and cell proliferation processes. One of them is HIF. A key component of cellular oxygen sensing is *HIF-1*, which activates a transcriptional program that promotes cell survival under insufficient oxygen supply conditions.^81^ It plays an important role in cellular and systemic oxygen homeostasis by regulating the expression of genes important for cell survival, glycolysis, erythropoiesis, catecholamine metabolism, etc.^82^ *HIF-1* is primarily regulated by changes in protein stability and transcriptional activity in an oxygen-dependent manner. Under these conditions, the normal level of oxygen observed in cells, HIF, rapidly decreases. In ischemic stroke, low tissue oxygenation occurs, and the activity of HIF inhibitory factors decreases (**Figure 1**), which leads to the stabilization of *HIF-1*, its accumulation inside cells and its translocation into the nucleus. This promotes the activation of the transcription of several dozen hypoxia-responsive genes.^83^ *HIF-1* activity is regulated by NO.^65^ *HIF-1* is an important component of changes in the tissue transcriptional repertoire during oxygen deficiency and plays a key role in regulating iNOS activity. *HIF-1*-dependent activation of eNOS transcription and the subsequent release of NO into the endothelium contributes to reducing the degree of ischemic stroke damage. Activation of HIF during hypoxia ensures optimal ATP production and cell integrity and is linked both directly and indirectly to the formation of reactive oxygen species.^84^ The balance between the production and elimination of reactive oxygen species and reactive nitrogen species leads to intracellular homeostasis, whereas their excessive formation leads to cell damage and, most likely, to changes in their metabolism.^85^ Changes in oxygen concentration affect the formation of reactive oxygen species. When their levels exceed the protective mechanisms, reactive oxygen species cause oxidative stress. Their elevated levels may also contribute to the stabilization of HIF during hypoxia and reoxygenation.

The level of NO, but not oxygen deficiency, reduces the angiogenic response during hypoxia by competitively inhibiting cytochrome C-oxidase.86 NO plays an important role in regulating oxygen delivery through paracrine control. Moreover, investigations of the interactions between oxygen (O_2_) and NO have led to the paradoxical conclusion that hypoxia leads to decreases in the expression and function of eNOS.^74^ Thus, the question arises as to why, during oxygen reduction in tissue, the activity of such a powerful vasodilator derived from the endothelium decreases. It appears that the functional interactions between NO and O_2_ are complex and poorly understood. Studies have demonstrated that HIF stabilization and transcriptional activity are achieved through two parallel pathways: first, by decreasing oxygen-dependent prolyl hydroxylation of HIF and, second, by NO-dependent S-nitrosylation of HIF pathway components. The necessity of these two complementary pathways of HIF activation arises from conditions in which a reduction in oxygen delivery can occur not only due to decreased oxygen levels in the environment but also because of decreased oxygen-carrying capacity of the blood, as in anemia.^87^ In turn, NO production is closely linked to O_2_ homeostasis. Oxygen is a key substrate for NO formation and affects the enzymatic activity and expression of enzymes that catalyze NO production. These relationships have been proven in a variety of clinical settings, ranging from the unique situation of humans living in hypoxic environments at high altitudes to the common scenario of anemia and conditions of therapeutics that can bind or release NO.

HIF has a heterodimeric structure consisting of HIFα and HIFβ subunits. The HIFα subunit is in the cytoplasm, and the β subunit is in the nucleus. The HIF-1β subunit is constitutively expressed. Three different isoforms of HIFα are known: HIF-1α, HIF-2α and HIF-3α. HIF-1α was the initial isoform identified by affinity purification using erythropoietin-derived oligonucleotides. HIF-2α and HIF-3α were discovered during the search for interaction partners for HIF-1α.^84^ HIF-1α is ubiquitously expressed and is a known regulator of hypoxic adaptation. It activates tissue survival pathways by inducing several key enzymes involved in cellular metabolism, angiogenesis and free radical scavenging. In response to hypoxia, HIF-1α is rapidly produced, stabilized, and transported to the nucleus. In contrast, it is rapidly degraded upon preoxygenation/reperfusion and under normal conditions.^65^ NO can modify hundreds of proteins within cells, including those involved in the regulation of HIF-1α.^80^ Short-term exposure to NO stabilized HIF-1α, whereas chronic exposure to NO destabilized HIF-1α.^88^

HIF-2α expression is tissue specific and restricted to specific cell types, such as vascular endothelial cells. HIF-1α plays a key role in the initial response to hypoxia, whereas HIF-2α is activated by chronic hypoxic exposure. Moreover, the degree of hypoxia required to induce each subtype varies.81 HIF-2α is induced at higher oxygen concentrations and for a longer time than HIF-1α.^89^ These data indicate that the two subtypes have complementary functions in the coordinated transcriptional response to hypoxia. Although HIFs were initially identified as oxygen-dependent transcription factors, recent studies have provided evidence for hypoxia-independent regulation of HIFs.^66^,^80^,^81^

Some studies have focused on the induction of microRNAs that positively or negatively influence the transcription of specific mRNAs.^81^,^90^ MicroRNAs are a class of endogenous tiny RNAs that inhibit translation or promote RNA degradation by forming a duplex within the untranslated region of mRNA. MicroRNAs play important roles in a wide range of cellular processes by fine-tuning gene expression.^81^ It has been shown that miR-155 promotes isoform-specific suppression of HIF-1α activity in cells exposed to prolonged hypoxia.^90^ Stabilization of HIF-1α during hypoxia leads to the expression of microRNA-210 and subsequently decreases mitochondrial respiration, which leads to a decrease in the production of reactive oxygen species.^91^ Activation of HIF helps maintain low levels of reactive oxygen species during hypoxia by suppressing the function of the mitochondrial tricarboxylic acid cycle. In other words, activation of HIF during hypoxia ensures optimal ATP production and cell integrity by minimizing reactive oxygen species during hypoxia.^84^

Systemic and brain-targeted HIF-1α stabilization may have different effects. Some findings suggest that the brain-targeted stabilization of HIF1α is a potential treatment for neonatal hypoxic–ischemic brain injury.^92^ HIFs are DNA-binding transcription factors that bind to specific nuclear cofactors during hypoxia. In humans, three genes encode different isoforms of HIF-α: *HIF1A*, encoding HIF-1α; endothelial per-aryl hydrocarbon receptor nuclear translocator-Sim (PAS) domain protein-1 or *HIF2A*, encoding HIF-2α; and *HIF3A*, encoding several splice variants of HIF-3α.^93^ HIF-1α is expressed ubiquitously, whereas HIF-2α, which was originally named endothelial PAS domain protein-1, is widely distributed in endothelial cells and some highly vascularized tissues. Thus, HIF-2α probably regulates endothelial-specific genes and has functions distinct from those of HIF-1α.^81^ HIFs are also associated with many diseases of the circulatory system. Considering that HIF pathways evolved to maintain oxygen homeostasis, it is not surprising that their disruption causes pathophysiological changes in the circulatory system. For example, diabetes or aging is a critical risk factor for ischemic heart disease, resulting in arterial obstruction due to an impaired response to ischemia. Therefore, controlling angiogenesis and arteriogenesis by modulating the HIF pathway may be valuable in patients with ischemic diseases.^81^ The role of *HIF-1* in ischemia remains unclear and controversial.^29^ *HIF-1* mediates genes both against and for neuronal survival in stroke.^94^ Exogenously administered NO metabolite S-nitrosoglutathione not only stabilizes *HIF-1* and induces *HIF-1*-dependent genes but also stimulates the regeneration process and promotes functional recovery in animals with brain injury. On the other hand, *HIF-1* regulates iNOS transcription and maintains iNOS translation during ischemia, thereby promoting ischemia-induced inflammation.^29^

## Nitric Oxide in Cerebral Ischemia

The participation of NO in the mechanisms underlying the development of various pathological conditions in the body has attracted great interest from researchers.^8^,^13^,^31^,^95^,^96^,^97^,^98^ During the development of ischemic damage, the regulatory systems of the brain, including the NO system, increase their activity. It is naturally accompanied by an increase in oxygen consumption and by the accumulation of underoxidized products in brain tissue.^99^,^100^,^101^ These processes are a consequence of hypoxia, which occurs after the disruption of the blood supply to organs and the development of oxygen deficiency.^74^ There is much evidence that a leading factor in the pathophysiological response of the brain to hypoxia-ischemia is the impairment of NO biosynthesis.^17^,^29^,^102^ Changes in NO content are also observed in a number of diseases, such as diabetes, cancer, Alzheimer’s disease and cardiovascular diseases.^22^,^75^,^100^ Many studies on the dynamics of NO in pathological processes have been conducted based on the measurement of NOS activity and EPR spectroscopy.^29^,^54^,^89^,^103^,^104^,^105^,^106^,^107^,^108^

Cerebral ischemia occurs when the blood supply to the brain decreases and oxygen deficiency occurs (**Figure 2**). It causes multiple and multidirectional changes in NO content in the brain, resulting in disturbances in signal transmission.^64^,^95^,^109^,^110^ In experiments, both the neurotoxic and neuroprotective properties of NO are recorded. To analyze the effects of changes in NO content, both donors of this substance and NOS inhibitors are used. The search for new classes of donors is in development.^52^,^58^,^111^,^112^ NOS activity measurements revealed that nNOS activity increased at the beginning of ischemia development,^113^ but iNOS expression was initiated within 24 hours after ischemia development.^106^ A sharp increase in NOS activity was also observed during the first hour of cerebral ischemia caused by occlusion of the middle cerebral artery.^103^ This increase occurred due to the activation of a large population of nNOS that was catalytically inactive under normal conditions. This finding demonstrated the tight functional regulation of physiological NO production in the brain.^114^ An increase in NO production over 60 minutes after ischemia was demonstrated by EPR spectroscopy, which revealed that NO production increased after 5 minutes of ischemia and continued for 60 minutes.^105^ Using the same method with the injection of a spin trap before ischemia, it was found that the relative concentration of free NO increased by 132% 15 minutes after ischemia caused by occlusion of the middle cerebral artery.^11^,^115^ This effect was reversible as NO levels gradually decreased over time and reached background levels after 60 minutes.^11^,^26^,^54^

**Figure 2:**
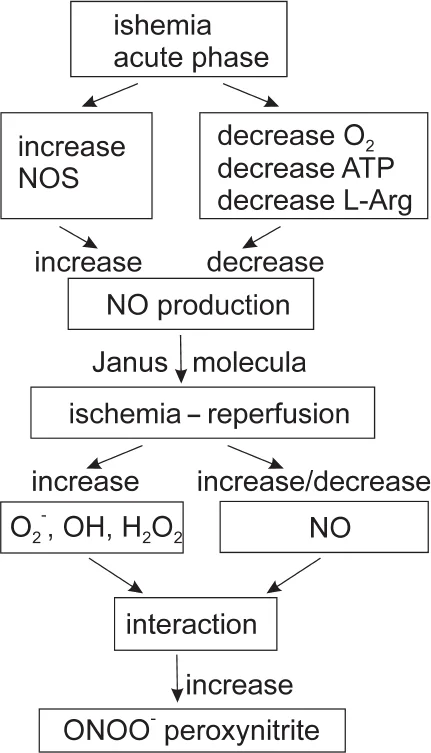
How NO is produced during the acute phase of ischemia–reperfusion. Cerebral ischemia occurs when the blood supply to the brain decreases and oxygen deficiency occurs. The main destructive effect of reperfusion is caused by the formation of highly reactive oxygen species such as O_2_-, OH, and H_2_O_2_. Created with CorelDRAW 2021. ATP: Adenosine triphosphate; H_2_O_2_: hydrogen peroxide; L-Arg: L-argenine; NO: nitric oxide; NOS: nitric oxide synthase; O_2_: oxygen; OH: hydroxyl radical; ONOO^−^: peroxynitrite.

The neurotoxic role of NO, which is generated during ischemia, has not been proven in some models of cerebral ischemia. This research revealed that the use of NOS inhibitors NG-nitro-l-arginine (L-NNA) and L-NG-Nitroarginine methyl ester (L-NAME) did not reduce the volume of infarction in a rat model of focal cerebral ischemia^116^,^117^ but, on the contrary, increased focal ischemic stroke.^118^ Numerous studies have investigated the use of NO donors as neuroprotective agents after short-term and permanent ischemic injury.^109^,^119^,^120^,^121^,^122^

In a study in which ischemic stroke was modeled by 5 minutes of hypobaric hypoxia (conditional ascent to an altitude of 4500 m above sea level) and an evaluation of NO content by EPR spectroscopy, the NO content in the hippocampus decreased 2–3-fold 5 hours after ischemic stroke was induced.^123^ This reduction in NO production persisted 24 and 72 hours after stroke. In another study in which focal stroke was modeled by irreversible coagulation of the left middle cerebral artery and the branch of the frontal vein crossing the middle cerebral artery, the average intensity of NO formation was 340 nM per hour/g of weight in the cortex of the right hemisphere and 395 nM per hour/g of weight in the cerebellum.^77^ Additionally, the content of NO in the spin trap in the ischemic part of the cortex of the left hemisphere decreased five -fold at 5 hours after ischemic stroke was induced, and this decrease persisted for 9, 24, and 72 hours after modeling.^77^ Moreover, the NO content in the spin trap in the cortex of the right hemisphere and the non-ischemic part of the cortex of the left hemisphere decreased by 40–50% 5, 9 and 24 hours after stroke induction. Interestingly, the content of NO in the R-conformer of hemoglobin did not change 5, 9 or 24 hours after stroke in the cortex of the right hemisphere or the non-ischemic part of the cortex of the left hemisphere compared with that in the intact control. The NO content of the R-conformer decreased in the cerebellum. The different dynamics of changes in the content of NO associated with hemoglobin were presumably due to different forms of NO deposition.^77^ In the literature, the R-conformer and T-conformer are usually interpreted as six- and five-coordinated heme iron complexes in hemoglobin.^77^ Perhaps the answer to this question lies in the compensatory change in the blood supply to these regions of the brain.

The accumulated contradictory information about NO, as a two-faced Janus, allowed us to state that, at present, there is no consensus on the role of endogenous NO in the processes occurring during damage to the nervous system.^5^,^55^,^78^,^112^,^122^,^124^ Nevertheless, this information is critical for choosing the optimal treatment strategy for brain damage.

## Nitric Oxide in Cerebral Ischemia–Reperfusion

I/R injury is a common pathological process in life-threatening conditions, such as shock, organ transplantation, and vascular surgery, where surgeons are required to occlude arteries. I/R injury occurs due to the influx of large amounts of oxygen during the reperfusion of ischemic tissues.^68^,^69^,^101^,^125^ The main destructive effect of reperfusion is caused by the formation of highly reactive oxygen species (**Figure 2**), such as O_2_- (superoxide), OH (hydroxyl radical) and H_2_O_2_ (hydrogen peroxide), which are formed in large quantities after the restoration of blood flow and recovery of oxygen supply.^55^,^69^,^125^ The role of NO during ischemia and in the I/R process is controversial due to the difficulty in measuring its concentration.^69^,^125^,^126^ NO is highly reactive and has a very short half-life. Measurements of nitrites and nitrates allow us to indirectly estimate the NO content. However, the most accurate and specific method for measuring NO is EPR.^51^,^54^,^59^,^77^ Under clinical conditions, I/R of the brain can be triggered by the formation of a thrombus in a cerebral artery, which leads to the disruption of metabolic processes. Perfusion can be successfully restored to the ischemic area, but brain damage during ischemia may be aggravated during reperfusion.^101^,^127^,^128^,^129^

Using the EPR spectroscopy method, we conducted an experimental analysis of the intensity of NO production in the olfactory bulb of the brain and hippocampus after cerebral ischemia was induced in rats. Modeling of cerebral ischemia was carried out by ligation at the level of the bifurcation of the common carotid arteries. Examples of EPR spectra of the olfactory bulb tissues in control rats and in rats 24 hours after ischemia induction are shown in **Figure 3**. We found that the content of NO in the olfactory bulb was significantly reduced to 47% in ischemic rats 24 hours after ischemia induction. The content of NO in the olfactory bulb of ischemic rats tended to decrease even more by 57% 2 days after ischemia.^79^,^130^

**Figure 3:**
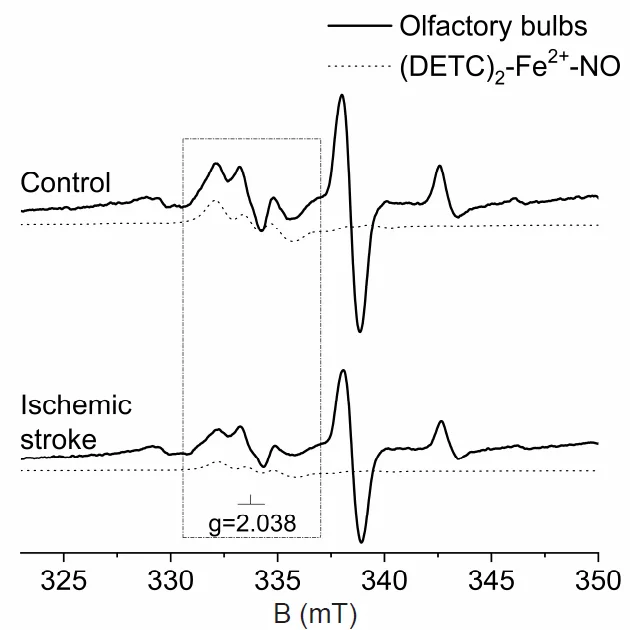
EPR spectra of olfactory bulb tissues from control rats and ischemic stroke rats 1 day after ischemia. The rats were injected with the (DETC)_2_–Fe^2+^ spintrap. The solid black lines show sample spectra, and the dashed lines display the contribution of the (DETC)_2_-Fe^2+^-NO signal in the sample spectrum. The frame shows the area of the signal (DETC)_2_-Fe^2+^-NO. The temperature was 77 K. The dotted line shows the contribution of NO to the observed signal. Created with CorelDRAW 2021. B: Magnitude of magnetic field (magnetic induction); DETC: diethyldithiocarbamate; EPR: electron paramagnetic resonance; NO: nitric oxide.

In experiments in which ischemia was induced by ligating the common carotid arteries at the bifurcation level, followed by the collection of 3 mL of blood from the common carotid artery, NO production in the hippocampus decreased by an average of 30%, and NO production in the frontal lobe tended to decrease one day after ischemia was induced.^97^,^98^ A comparative experimental analysis of the intensity of NO production in the hippocampal tissues using EPR spectroscopy in rats after hemorrhagic stroke was induced by microinjecting 500 nL of autologous blood into the brain to a depth of 5.0 mm (hippocampus) on the left side. NO content in the hippocampus reliably decreased by 36% on the 3^rd^ day after hemorrhagic stroke was induced.^79^

## Therapeutic Potential of Nitric Oxide

### Role of nitric oxide synthase in the development of ischemic damage

As stated earlier, nNOS dimerization is a prerequisite for NO synthesis. Disruption of dimerization, as well as a deficiency or absence of L-arginine, leads to the initial formation of superoxide anions and peroxynitrite, which are catalyzed by the nNOS monomer.^23^,^32^ Although superoxide anions may perform an important signaling function in some tissues, the formation of reactive oxygen species has a predominantly negative effect and underlies the development of some neurodegenerative diseases. Impaired eNOS dimerization, which occurs in cases of S-nitrosylation, leads to a decrease in its enzymatic activity. In turn, a decrease in eNOS enzymatic activity is a possible cause of endothelial dysfunction, which is observed in age-related changes in blood vessels and hypertension.^17^

Activation of nNOS synthesis is most often an adaptive response to any damage to nervous tissue, for example, during strokes observed after traumatic injury.^38^ This increase in nNOS is thought to mediate cell death processes. The main processes of cell death triggered by ischemic damage to nervous tissue are apoptosis (a strictly regulated process of programmed cell death) and necrosis (cell death that occurs during the disruption of the integrity of the cell membrane). The main factors triggering these processes are NMDA-induced excitotoxicity and the formation of reactive oxygen species associated with an increase in the amount of NO.^29^

Early mechanisms of ischemic injury development are mainly associated with a calcium-dependent increase in nNOS activity, whereas late mechanisms are characterized by iNOS expression several hours after injury.^114^ Increased nNOS activity in the early stage of ischemia leads to an excessive increase in NO content, which, in turn, leads to necrotic death and the formation of reactive oxygen and nitrogen species through the S-nitrosylation reaction. Reactive oxygen and nitrogen species trigger secondary ischemic changes, including apoptosis. NO itself is capable of reacting with superoxide radicals to form peroxynitrite. It is an extremely aggressive compound that can inactivate or destroy most cellular proteins, even at moderate doses, disrupting cellular signaling pathways and inducing apoptosis and necrosis.^32^,^69^

In the late stages of ischemia, iNOS is activated as a result of the migration of immune cells and the release of inflammatory mediators. A subsequent increase in NO concentration leads to the distribution of neurotoxicity and to apoptosis and necrosis of neighboring cells of the nervous tissue, glia and endothelium of adjacent vessels.^29^,^78^,^124^ Similar changes are observed during reperfusion of ischemic tissues; the activation of iNOS increase the number of immune cells and mediators, which appear as a result of reperfusion, and the subsequent increase in NO continues the destruction of surrounding tissues, including glia and endothelium, which leads to a disruption of the integrity of the blood–brain barrier.^69^

It is assumed that in the processes of ischemic damage, eNOS plays a protective role: it not only causes the expansion of local vessels but also increases the proliferation and migration of smooth muscle cells of the vascular wall, which is important for the restoration of the bloodstream during the process of recovery after ischemia.^29^,^34^

### Therapeutic potential of endogenous nitric oxide

Currently, the continuous production of endogenous NO, which acts as a universal regulator or as a signaling molecule in various biochemical and physiological processes, is detected in animal and human tissues and in plants and bacteria.^1^,^20^,^51^ In the nervous system, NO acts as an unconventional neurotransmitter to mediate neuronal signaling, since NO is a gaseous molecule that is not stored in synaptic vesicles and is synthesized by neurons in response to an incoming signal.^2^ NO diffuses from the cells in which it is synthesized and reaches neighboring cells, causing a response. Despite its instability, NO can function as an endogenous vasodilator by regulating vascular tone and can perform a number of other functions.^4^,^17^,^20^ Moreover, NO is a radical that can react with various biological substrates both by nitrosylation and nitrosation. Nitrosylation occurs as the direct reaction of NO with a reagent, whereas nitrosation occurs as the indirect transfer of NO to biological nucleophiles via previously formed NO^+^ due to NO oxidation.^3^,^18^,^131^

NO is a highly diffuse gas that acts as an intercellular messenger and is widespread in the nervous system. NO is assumed to play a role in synaptic plasticity, and several studies have indicated the importance of NO signaling in memory.^30^ However, the lifetime of NO is short for all observed functions.^11^,^18^,^62^,^132^ In biological systems, the chemical reactivity of NO is relatively limited to reactions with metalloproteins (heme-containing proteins) and with other free radicals. While this seems limited, these reactions may involve the ligation of metals with a concomitant change in protein structure and activity and may involve the oxidation or reduction of NO and the formation of various secondary species with unique biochemical properties and reactivities that collectively may facilitate oxidative, nitrosative, or nitrative reactions.^48^,^51^

Due to the high affinity of NO for heme, heme-containing proteins are common targets of NO activity. The key reaction responsible for vasodilation is binding of NO with soluble guanylyl cycles, followed by the formation of cGMP.^4^,^17^,^29^,^61^,^133^,^134^

The interaction of NO with iron in heme leads to abrupt changes in the biochemical activity of heme-containing proteins. Therefore, iron in heme is considered the main target of the biological action of NO.^48^,^57^,^68^ NO widely interacts not only with heme but also with thiol-containing proteins, which interact with NO^+^ to form S-nitrosothiols (RS-NO).^55^

Notably, the high-speed reaction of free NO with superoxide, which is defined by the speed of diffusion, leads to the conversion of NO in cells and tissues to a highly toxic substance, peroxinitrite. Thus, many researchers agree that the function of NO, especially as a paracrine effector, is provided by the formation of chemical NO, which contains compounds that are able to protect NO from the destructive effect of superoxide and to deliver it to its targets.^11^,^26^,^111^ Examples of such compounds include S-nitrosothiols and dinitrosyl iron complexes (DNICs) with low- and high-molecular-weight thiol-containing ligands (**Figure 4**), which provide the delivery of NO to the corresponding targets of its biological activity.^48^,^111^,^134^,^135^,^136^ Small molecules such as S-nitrosothiols and DNICs can serve as alternative methods for the storage and delivery of NO.^48^,^111^,^134^,^137^,^138^ In living organisms, RS-NO and DNICs can mutually transform into each other, which makes them interdependent. Nevertheless, DNICs play a decisive role in the DNIC-RS-NO system, as DNICS determines the emergence of this system itself.^111^,^139^

**Figure 4:**
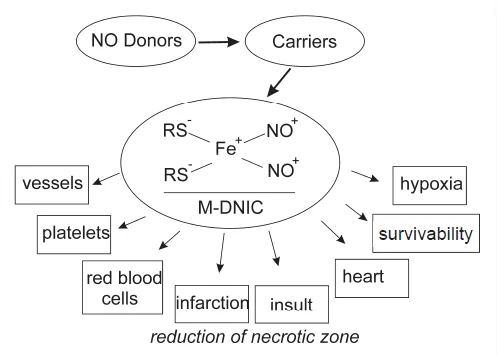
How DNIC complexes act on various tissues. Created with CorelDRAW 2021. DNICs: Dinitrosyl iron complexes; M-DNICs: mononuclear DNICs; NO: nitric oxide.

At present, DNICs are considered the main form of stored NO in living organisms (**Figure 4**). DNICs exhibit a wide range of biological effects, including potent vasodilatory and hypotensive actions, inhibition of thrombocyte aggregation, anti-hypoxic protection of the myocardium, enhancement of red blood cell elasticity, promotion of skin wound healing, improvement of survival after hemorrhage, reduction in the necrotic area following myocardial infarction, suppression of programmed cell death, modulation of gene expression, inhibition of early-stage tumor growth, and induction of erection (**Figure 4**).^111^

GSH, cysteine, and histidine can function as ligands for low-molecular-weight DNICs, and amino acid residues can function as ligands for high-molecular-weight DNICs or protein DNICs. These results significantly change our understanding of the role of NO in the function of biological systems. They describe the possibility of NO distribution in areas where synthesis of NO is not observed and explain the prolonged effects of NO.

DNICs are considered relatively stable forms of NO in living cells. They serve as one of the most important depots of NO and as a mechanism of NO delivery to the binding site.^12^,^23^,^46^,^48^,^51^,^134^ Thus, we discuss DNICs as a family of DNICs with thiol-containing ligands (RS^–^). The implementation of various metabolic and physiological processes, i.e., biological systems of NO, is possible.^111^,^140^ However, the role of DNICs as a working or active form of NO is possible only when the molecule, which ensures the transfer of NO and NO^+^ to their targets, is sufficiently stable. Such stability is achieved by including thiol-containing ligands in DNICs-RS, which provide electron density transfer to NO^+^ and prevent the hydrolysis of these cations and stabilize DNICs-RS.^19^,^137^,^141^ The idea of creating the donors of NO based on different variations of DNICs seems advantageous. However, there is some complexity in solving this task: under natural conditions, DNICs are unstable, and it is very difficult to isolate them. Nevertheless, the authors of these studies managed to create a medicine, Ohakam.^142^,^143^

On the other hand, research on the biological activity of various DNIC complexes has shown that the efficiency of action of DNICs-RS depends on the nature of the thiol-containing ligands. Therefore, DNICs with L-cysteine induce rapid, intensive vasodilatory responses, and more stable DNICs with GSH are characterized by slow, long-term effects.^141^ In living objects, they represent two forms: mononuclear DNICs (M-DNICs) and binuclear DNICs (B-DNICs), characterized by chemical formulas [(RS^–^)_2_Fe^+^(NO^+^)_2_]) and [(RS^–^)_2_ Fe^2+^(NO^+^)_4_], respectively.^3^,^51^,^111^ In animal tissues *in vivo*, DNICs with thiol-containing ligands, which are injected into animal tissue or produced with the participation of endogenous NO, are presented mainly as diamagnetic B-DNICs. This form was not detected by the EPR method. M-DNICs with thiol-containing ligands are paramagnetic with S = ½.^58^,^144^

Based on the results given above, it is clear that different DNIC complexes can be applied with different aims. Spray containing B-DNIC-GSH (4 mmol/cm^2^) decreased inflammation and promoted fibroblast proliferation, angiogenesis and growth of granulation tissue at the 4^th^ day after injury. The use of a spray containing NO-CGF (1 mmol/cm^2^) decreased inflammation and promoted fibroblast proliferation, angiogenesis and growth of granulation tissue within 4 days after injury.^145^ Additionally, injection of B-DNIC-GSH into the rat blood stream before hemorrhage significantly protected heart function under hemorrhagic shock conditions, which increased the stroke volume and cardiac output of the left ventricle^146^ by 1.5 times compared with the control values. Preliminary use of DNIC-GSH significantly (up to 21%) increased survival under conditions of hemorrhagic shock. It is assumed that the beneficial effect of DNIC-GSH on the parameters of systemic circulation during hemorrhagic shock is determined by its antioxidant activity and ability to induce S-nitrosylation of proteins.^146^

The hypotensive effect of DNIC-GSH has been well studied.^14^ It was demonstrated that DNIC-GSH is a medicament with powerful hypotensive effects and is used for the relief of hypertensive crisis. Single intravenous administration of it induced a decrease in blood pressure in just 3–4 minutes and then resulted in slow recovery for 8–10 hours.^142^ It was found that medicaments based on DNIC-GSH retained high vasodilator and hypotensive activity for 15 years when stored at room temperature in the dark.^142^,^143^ Several studies have investigated the antioxidant effects of DNIC in various model systems. DNIC-GSH effectively inhibits Cu^2+^-induced peroxidation of low-density lipoprotein. Moreover, DNIC-GSH suppresses the formation of reactive oxygen species during the co-oxidation of lecithin liposomes and glucose. Additionally, DNIC-GSH carried superoxide radical (O_2_·^−^) generated in the xanthine-xanthine oxidase system.^135^,^138^ Recent studies have substantiated the antioxidant activity of NO-containing iron complexes and proposed their use as promising pharmacological agents.

Although this section is devoted to the therapeutic potential of DNICs, it should be noted that NO is a Janus molecule.^76^,^124^ NO can have either neuroprotective or neurotoxic effects. The direction of its activity depends on many factors, including its amount, type of activated NOS for its production, and type of targeting receptors. These experiments are discussed in the other sections. To illustrate the difficult answers to these questions, we provide an example of our research in another area. We showed that keeping the animals in conditions of limited physical activity for 1 month led to a 2–3-fold increase in the NO concentration in heart tissue.^147^

The results of the latest studies of DNICs with thiol-containing ligands show that these compounds can be implemented not only as donors of neutral NO molecules but also as donors of NO^+^.^111^,^148^ This ability can be responsible for the cytotoxic activity of DNIC complexes. The available results indicate that NO^+^ has a toxic effect on cell cultures.^149^,^150^ This ability of DNICs may be responsible for the cytotoxic activity of these complexes. The available results indicate that NO^+^ has a toxic effect on cell cultures.^148^,^150^

Thus, DNIC complexes can function as donors of NO molecules, which are responsible for the positive regulatory effects of the NO system on physiological and biochemical processes, as well as NO^+^, which is involved in the negative cytotoxic activity of the NO system.^111^ Some researchers have reported that DNICs are donor NO, but NO is not spontaneously released from complexes with donors and requires the action of a factor that destroys the complex.^151^

As a result of numerous investigations, Professor A. F. Vanin summarized these results: the inclusion of NO molecules in these complexes (DNICs) ensures its stabilization and transfer of NO both inside and outside cells and tissues to targets of biological action and its oxidized form NO^+^ to heme-containing proteins, iron-sulfur and thiol-containing proteins.^55^ Therefore, DNIC-GSH can serve as a basis for the generation of medicaments with a variety of therapeutic effects.

### Neurotoxic and neuroprotective effects of nitric oxide

On the one hand, the development of cerebral ischemia and the subsequent occurrence of stroke are associated with disturbances in cerebral blood flow, as well as with disturbances in the regulation of the blood supply of the brain by the NO system.^64^,^75^,^95^,^152^ On the other hand, hypoxia resulting from ischemic stroke is accompanied by early cell death in various parts of the brain and subsequent programmed apoptotic death of other cells.^68^,^110^

Disruption of the oxygen supply of the brain also occurs when a blood clot forms in a blood vessel or when an aneurysm ruptures, which often results in ischemic or hemorrhagic stroke.^22^,^75^ As mentioned above, the role of NO in these hypoxia-ischemia processes is contradictory: NO is capable of performing both neurotoxic and neuroprotective effects.^22^,^102^,^121^,^149^ With the development of pathological processes in the brain (hypoxia and ischemia), the regulatory systems of the brain (including the NO system) increase activity, which is naturally accompanied by an increase in oxygen consumption (which aggravates hypoxia) and an increase in underoxidized products in brain tissue.^22^,^102^,^121^,^153^ The function of the NO system is disrupted during hypoxia and cerebral ischemia, and cerebral ischemia is accompanied by multiple and multidirectional changes in the NO content in the brain and disturbances in the transmission of signals between cells.^64^,^96^,^122^

Notably, researchers use different models of ischemia depending on the objectives of the study and which develop according to different mechanisms. First, this concerns the expression of different types of NOS. Most authors agree that the main functions of NO, as a signaling mechanism regulating all critical cellular functions, are provided by eNOS.^12^,^32^,^69^,^75^,^154^ eNOS activation facilitates recovery from ischemic injury.^150^,^151^ Evidence about the role of nNOS in the development of cerebral ischemia is contradictory.^2^,^22^,^25^,^96^

Thus, on the other hand, it is believed that neuronal NO production aggravates acute ischemic injury, whereas vascular NOS (eNOS) plays a protective role after middle cerebral artery occlusion. Other authors have indicated that NO overexpression begins with the activation of a large population of nNOS.^25^,^96^,^114^ NO generated by iNOS has the potential to be a pathological source of NO, leading to the overexpression of neurotransmitters and disruption of normal physiological processes.^6^,^22^ For this reason, blocking the activity of nNOS and iNOS during the development of hypoxia is recommended to prevent cerebral ischemia. However, these recommendations are conditional and archaic to a certain extent, since many other neurotransmitter systems and trophic factors present in the brain are also involved in the processes of stabilizing brain functions during hypoxia and ischemia.

Hypoxic or ischemic stress can cause many serious brain injuries, including stroke and neonatal hypoxic ischemic encephalopathy. During cerebral hypoxia and ischemia, NO plays either a neurotoxic or neuroprotective role, depending on factors such as the NOS isoform, the type of cells that produce NO, the time period after the beginning of hypoxic-ischemic brain injury, the characteristics of the blood supply to the brain, the age of the patient, the adequacy of the therapy, and many other factors. During I/R, NO also has dual effects: neuroprotection and neurotoxicity. Excessive NO production can be neurotoxic if it leads to excitotoxic cascades, inflammation, and apoptosis and worsens primary brain injury. In contrast, NO, produced by eNOS, plays a neuroprotective role by maintaining cerebral blood flow, preventing neuronal injury, and inhibiting platelet and leukocyte adhesion. NO induced by NOS derivatives and nNOS may also play a neuroprotective role in specific brain regions.^22^,^29^,^96^ The effectiveness of the search for neuroprotective mechanisms in cerebral ischemia is also evidenced by a phenomenon such as preconditioning.^68^,^72^,^126^

The significant role of NO in many functional processes in the body under normal or pathological conditions, including the activity of the nervous system, as well as the lack of information about the amount of synthesized NO, predetermines the importance of further research in this area. Therefore, the solution to the problem of the dynamics of NO production in tissues during the development of various processes, including pathological processes, seems extremely relevant and contributes to the development of new approaches to the pharmacological correction of emerging disorders.

## Conclusion

Currently, the idea that NO plays a multifaceted role in biological processes is unclear. The main function of NO is vasodilation, which controls blood vessel tone and oxygen delivery to tissues. NO is also a neuromodulator and neurotransmitter and performs many other functions. At the initial stage of its investigation, when it became clear that the lifetime of NO in the body is short, its functions did not seem very extensive. However, as the mechanisms of its action have been widely studied, it has become known that NO has a large depot. This is the way NO exerts its regulatory and modulating effects. The significant role of NO in many functional processes in the body, in normal and pathological conditions, including in the activity of the nervous system, as well as the lack of information on the amount of NO synthesized and its functions in various pathologies, predetermine the importance of further research in this area. Therefore, the solution to the issue of the dynamics of NO production in tissues during the development of various processes, including pathological processes, seems extremely relevant and contributes to the development of new approaches to the pharmacological correction of emerging disorders.

**Facts**

1. Nitric oxide is a highly chemically reactive molecule that can function as both an oxidizing agent and a reducing agent, leading to diverse biological effects.
2. The key physiological functions of nitric oxide include vasodilation (widening blood vessels) and modulation of cellular energy metabolism.
3. Nitric oxide plays a central role in the body’s molecular mechanisms in response to hypoxia (low oxygen conditions).

**Open Questions**

1. How exactly does nitric oxide participate in and regulate long-term adaptation to hypoxia, and how do its signaling pathways interact with other hypoxia-induced factors?
2. Can the nitric oxide pathway be therapeutically targeted to improve outcomes in ischemic cerebrovascular disease, and if so, should the aim be to enhance or inhibit its signaling at different disease stages?
3. How does the dual chemical nature of nitric oxide (as both an oxidant and reductant) dictate its functional outcome in the complex and evolving tissue environment following a cerebrovascular event?

## Data Availability

*No additional data are available.*

## References

[1] Leonid LM (2001). Gaseous transmission across time and species. Am Zool.

[2] Garthwaite J (2019). NO as a multimodal transmitter in the brain: discovery and current status. Br J Pharmacol.

[3] Vanin AF (2021). Physico-chemistry of dinitrosyl iron complexes as a determinant of their biological activity. Int J Mol Sci.

[4] Lundberg JO, Weitzberg E (2022). Nitric oxide signaling in health and disease. Cell.

[5] Guix FX, Uribesalgo I, Coma M, Muñoz FJ (2005). The physiology and pathophysiology of nitric oxide in the brain. Prog Neurobiol.

[6] Steinert JR, Chernova T, Forsythe ID (2010). Nitric oxide signaling in brain function, dysfunction, and dementia. Neuroscientist.

[7] Santos RM, Lourenço CF, Ledo A, Barbosa RM, Laranjinha J (2012). Nitric oxide inactivation mechanisms in the brain: role in bioenergetics and neurodegeneration. Int J Cell Biol.

[8] Reutov VP, Okhotin VE, Shuklin AV, Sorokina EG, Kositsyn NS, Gurin VN (2007). Nitric oxide (NO) and NO cycle in myocardium: molecular, biochemical and physiological aspects. Usp Fiziol Nauk.

[9] Signori D, Magliocca A, Hayashida K (2022). Inhaled nitric oxide: role in the pathophysiology of cardio-cerebrovascular and respiratory diseases. Intensive Care Med Exp.

[10] Kamenshchikov NO, Duong N, Berra L (2023). Nitric oxide in cardiac surgery: a review article. Biomedicines.

[11] Tennyson AG, Lippard SJ (2011). Generation, translocation, and action of nitric oxide in living systems. Chem Biol.

[12] Cortese-Krott MM, Kramer CM, Kelm M (2017). NOS, NO, and the red cell. Nitric Oxide: Elsevier.

[13] Manukhina EB, Tseilikman VE, Karpenko MN (2020). Intermittent hypoxic conditioning alleviates post-traumatic stress disorder-induced damage and dysfunction of rat visceral organs and brain. Int J Mol Sci.

[14] Lakomkin VL, Vanin AF, Timoshin AA, Kapelko VI, Chazov EI (2007). Long-lasting hypotensive action of stable preparations of dinitrosyl-iron complexes with thiol-containing ligands in conscious normotensive and hypertensive rats. Nitric Oxide.

[15] Adrianov VV, Sitdikov FG, Gaĭnutdinov Kh L (2008). Changes in nitric oxide in heart of intact and sympathectomized rats of different age. Ontogenez.

[16] Luiking YC, Engelen MP, Deutz NE (2010). Regulation of nitric oxide production in health and disease. Curr Opin Clin Nutr Metab Care.

[17] Chachlaki K, Prevot V (2020). Nitric oxide signalling in the brain and its control of bodily functions. Br J Pharmacol.

[18] Heinrich TA, da Silva RS, Miranda KM, Switzer CH, Wink DA, Fukuto JM (2013). Biological nitric oxide signalling: chemistry and terminology. Br J Pharmacol.

[19] Vanin AF (2019). What is the mechanism of nitric oxide conversion into nitrosonium ions ensuring s-nitrosating processes in living organisms. Cell Biochem Biophys.

[20] Ignarro LJ, Cirino G, Casini A, Napoli C (1999). Nitric oxide as a signaling molecule in the vascular system: an overview. J Cardiovasc Pharmacol.

[21] Förstermann U, Sessa WC (2012). Nitric oxide synthases: regulation and function. Eur Heart J.

[22] Liu H, Li J, Zhao F, Wang H, Qu Y, Mu D (2015). Nitric oxide synthase in hypoxic or ischemic brain injury. Rev Neurosci.

[23] Andrew PJ, Mayer B (1999). Enzymatic function of nitric oxide synthases. Cardiovasc Res.

[24] Li H, Poulos TL (2005). Structure-function studies on nitric oxide synthases. J Inorg Biochem.

[25] Castro Faria Neto H, Reis P, Maron-Gutierrez T, Gonçalves-De-Albuquerque CF, Silva A, Saeedi Saravi SS (2017). Role of nitric oxide synthase in the function of the central nervous system under normal and infectious conditions. Nitric oxide synthase - simple enzyme-complex roles.

[26] Hill BG, Dranka BP, Bailey SM, Lancaster JR, Darley-Usmar VM (2010). What part of NO don’t you understand? Some answers to the cardinal questions in nitric oxide biology. J Biol Chem.

[27] Lundberg JO, Weitzberg E, Ignarro LJ, Freeman BA (2017). Chapter 12 - Nitric oxide formation from inorganic nitrate. Nitric oxide.

[28] Lee SJ, Stull JT (1998). Calmodulin-dependent regulation of inducible and neuronal nitric-oxide synthase. J Biol Chem.

[29] Wierońska JM, Cieślik P, Kalinowski L (2021). Nitric Oxide-dependent pathways as critical factors in the consequences and recovery after brain ischemic hypoxia. Biomolecules.

[30] Argyrousi EK, Prickaerts J, Ennaceur A, de Souza Silva MA (2018). Chapter 37 - Nitrinergic signalling in object novelty recognition. Handbook of behavioral neuroscience.

[31] Panda K, Ghosh S, Stuehr DJ (2001). Calmodulin activates intersubunit electron transfer in the neuronal nitric-oxide synthase dimer. J Biol Chem.

[32] Pacher P, Beckman JS, Liaudet L (2007). Nitric oxide and peroxynitrite in health and disease. Physiol Rev.

[33] Dudzinski DM, Michel T (2007). Life history of eNOS: partners and pathways. Cardiovasc Res.

[34] Cui X, Chopp M, Zacharek A, Zhang C, Roberts C, Chen J (2009). Role of endothelial nitric oxide synthetase in arteriogenesis after stroke in mice. Neuroscience.

[35] Godo S, Shimokawa H (2017). Divergent roles of endothelial nitric oxide synthases system in maintaining cardiovascular homeostasis. Free Radic Biol Med.

[36] Venema RC (2002). Post-translational mechanisms of endothelial nitric oxide synthase regulation by bradykinin. Int Immunopharmacol.

[37] Hayashi Y, Nishio M, Naito Y (1999). Regulation of neuronal nitric-oxide synthase by calmodulin kinases. J Biol Chem.

[38] Christopherson KS, Bredt DS (1997). Nitric oxide in excitable tissues: physiological roles and disease. J Clin Invest.

[39] Mungrue IN, Bredt DS (2004). nNOS at a glance: implications for brain and brawn. J Cell Sci.

[40] Stamler JS, Meissner G (2001). Physiology of nitric oxide in skeletal muscle. Physiol Rev.

[41] Balke JE, Zhang L, Percival JM (2019). Neuronal nitric oxide synthase (nNOS) splice variant function: Insights into nitric oxide signaling from skeletal muscle. Nitric Oxide.

[42] Casadei B (2006). The emerging role of neuronal nitric oxide synthase in the regulation of myocardial function. Exp Physiol.

[43] Zhang YH, Jin CZ, Jang JH, Wang Y (2014). Molecular mechanisms of neuronal nitric oxide synthase in cardiac function and pathophysiology. J Physiol.

[44] Chi OZ, Liu X, Weiss HR (2003). Effects of inhibition of neuronal nitric oxide synthase on NMDA-induced changes in cerebral blood flow and oxygen consumption. Exp Brain Res.

[45] Ichihara A, Inscho EW, Imig JD, Navar LG (1998). Neuronal nitric oxide synthase modulates rat renal microvascular function. Am J Physiol.

[46] Leone AM, Palmer RM, Knowles RG, Francis PL, Ashton DS, Moncada S (1991). Constitutive and inducible nitric oxide synthases incorporate molecular oxygen into both nitric oxide and citrulline. J Biol Chem.

[47] Béchade C, Colasse S, Diana MA, Rouault M, Bessis A (2014). NOS2 expression is restricted to neurons in the healthy brain but is triggered in microglia upon inflammation. Glia.

[48] Patel RP, Yuan S, Kevil CG, Ignarro LJ, Freeman BA (2017). Chapter 4 - S-Nitrosothiols and nitric oxide biology. Nitric oxide.

[49] Jensen FB (2009). The role of nitrite in nitric oxide homeostasis: a comparative perspective. Biochim Biophys Acta.

[50] Maia LB, Moura JJ (2011). Nitrite reduction by xanthine oxidase family enzymes: a new class of nitrite reductases. J Biol Inorg Chem.

[51] Vanin AF (2016). Dinitrosyl iron complexes with thiol-containing ligands as a “working form” of endogenous nitric oxide. Nitric Oxide.

[52] Grachev DI, Shumaev KB, Kosmachevskaya OV, Topunov AF, Ruuge EK (2021). Nitrosyl comlexes of hemoglobin in various model systems. Biophysics.

[53] Wink DA, Mitchell JB (1998). Chemical biology of nitric oxide: Insights into regulatory, cytotoxic, and cytoprotective mechanisms of nitric oxide. Free Radic Biol Med.

[54] Jakubowska MA, Pyka J, Michalczyk-Wetula D (2020). Electron paramagnetic resonance spectroscopy reveals alterations in the redox state of endogenous copper and iron complexes in photodynamic stress-induced ischemic mouse liver. Redox Biol.

[55] Vanin AF (2017). Dinitrosyl iron complexes with thiol-containing ligands as a base for developing drugs with diverse therapeutic activities: Physicochemical and biological substantiation. Biophysics.

[56] Su H, Liu X, Du J, Deng X, Fan Y (2020). The role of hemoglobin in nitric oxide transport in vascular system. Med Nov Technol Devices.

[57] Hoiland RL, MacLeod DB, Stacey BS (2023). Hemoglobin and cerebral hypoxic vasodilation in humans: Evidence for nitric oxide-dependent and S-nitrosothiol mediated signal transduction. J Cereb Blood Flow Metab.

[58] Borodulin RR, Kubrina LN, Mikoyan VD (2013). Dinitrosyl iron complexes with glutathione as NO and NO^+^ donors. Nitric Oxide.

[59] Mikoyan VD, Burgova EN, Borodulin RR, Vanin AF (2020). Dinitrosyl iron complexes with thiol-containing ligands exist in living organisms mainly in the binuclear form. Biophysics.

[60] Nakane M (2003). Soluble guanylyl cyclase: physiological role as an NO receptor and the potential molecular target for therapeutic application. Clin Chem Lab Med.

[61] Koesling D, Russwurm M, Mergia E, Ignarro LJ, Freeman BA (2017). Chapter 8 - Regulation and physiological functions of NO-sensitive guanylyl cyclase. Nitric oxide.

[62] Thomas DD, Ridnour LA, Isenberg JS (2008). The chemical biology of nitric oxide: implications in cellular signaling. Free Radic Biol Med.

[63] Liu C, Liang MC, Soong TW (2019). Nitric Oxide, Iron and Neurodegeneration. Front Neurosci.

[64] Manukhina EB, Malyshev IY, Smirin BV, Mashina SY, Saltykova VA, Vanin AF (1999). Production and storage of nitric oxide in adaptation to hypoxia. Nitric Oxide.

[65] Khan M, Khan H, Singh I, Singh AK (2017). Hypoxia inducible factor-1 alpha stabilization for regenerative therapy in traumatic brain injury. Neural Regen Res.

[66] Chen H, Ma D, Yue F (2022). The potential role of hypoxia-inducible factor-1 in the progression and therapy of central nervous system diseases. Curr Neuropharmacol.

[67] Donnan GA, Fisher M, Macleod M, Davis SM (2008). Stroke. Lancet.

[68] Ivanov EV, Gavrilova SA, Koshelev VB (2021). Brain acute ischemia mechanisms: implications to experimental and clinical treatment. Reg Blood Circ Microcirc.

[69] Wang Y, Hong F, Yang S (2022). Roles of nitric oxide in brain ischemia and reperfusion. Int J Mol Sci.

[70] Lambertsen KL, Biber K, Finsen B (2012). Inflammatory cytokines in experimental and human stroke. J Cereb Blood Flow Metab.

[71] Martin RH, Yeatts SD, Hill MD, Moy CS, Ginsberg MD, Palesch YY (2016). ALIAS (albumin in acute ischemic stroke) trials: analysis of the combined data from parts 1 and 2. Stroke.

[72] Deryagin OG, Gavrilova SA, Gainutdinov KL (2017). Molecular bases of brain preconditioning. Front Neurosci.

[73] Capizzi A, Woo J, Verduzco-Gutierrez M (2020). Traumatic brain injury: an overview of epidemiology, pathophysiology, and medical management. Med Clin North Am.

[74] Umbrello M, Dyson A, Feelisch M, Singer M (2013). The key role of nitric oxide in hypoxia: hypoxic vasodilation and energy supply-demand matching. Antioxid Redox Signal.

[75] Garry PS, Ezra M, Rowland MJ, Westbrook J, Pattinson KT (2015). The role of the nitric oxide pathway in brain injury and its treatment--from bench to bedside. Exp Neurol.

[76] Calabrese V, Cornelius C, Rizzarelli E, Owen JB, Dinkova-Kostova AT, Butterfield DA (2009). Nitric oxide in cell survival: a Janus molecule. Antioxid Redox Signal.

[77] Gainutdinov KL, Gavrilova SA, Iyudin VS (2011). EPR study of the intensity of the nitric oxide production in rat brain after ischemic stroke. Appl Magn Reson.

[78] Maggio DM, Singh A, Iorgulescu JB (2017). Identifying the long-term role of inducible nitric oxide synthase after contusive spinal cord injury using a transgenic mouse model. Int J Mol Sci.

[79] Andrianov VV, Kulchitsky VA, Yafarova GG (2021). Comparative study of the intensity of nitric oxide production and copper content in hippocampus of rats after modeling of hemorrhagic stroke and brain injury. Appl Magn Reson.

[80] Hendrickson MD, Poyton RO (2015). Crosstalk between nitric oxide and hypoxia-inducible factor signaling pathways: an update. Res Rep Biochem.

[81] Hashimoto T, Shibasaki F (2015). Hypoxia-inducible factor as an angiogenic master switch. Front Pediatr.

[82] Helton R, Cui J, Scheel JR (2005). Brain-specific knock-out of hypoxia-inducible factor-1alpha reduces rather than increases hypoxic-ischemic damage. J Neurosci.

[83] Manalo DJ, Rowan A, Lavoie T (2005). Transcriptional regulation of vascular endothelial cell responses to hypoxia by HIF-1. Blood.

[84] Chen R, Lai UH, Zhu L, Singh A, Ahmed M, Forsyth NR (2018). Reactive oxygen species formation in the brain at different oxygen levels: the role of hypoxia inducible factors. Front Cell Dev Biol.

[85] Shlapakova TI, Kostin RK, Tyagunova EE (2020). Reactive oxygen species: participation in cellular processes and progression of pathology. Russ J Bioorg Chem.

[86] Arce C, Vicente D, Monto F (2021). Hypoxia increases nitric oxide-dependent inhibition of angiogenic growth. Int J Transl Med.

[87] Jeffrey Man HS, Tsui AK, Marsden PA (2014). Nitric oxide and hypoxia signaling. Vitam Horm.

[88] Huang LE, Willmore WG, Gu J, Goldberg MA, Bunn HF (1999). Inhibition of hypoxia-inducible factor 1 activation by carbon monoxide and nitric oxide. Implications for oxygen sensing and signaling. J Biol Chem.

[89] Wiesener MS, Jürgensen JS, Rosenberger C (2003). Widespread hypoxia-inducible expression of HIF-2alpha in distinct cell populations of different organs. FASEB J.

[90] Cho H, Ahn DR, Park H, Yang EG (2007). Modulation of p300 binding by posttranslational modifications of the C-terminal activation domain of hypoxia-inducible factor-1alpha. FEBS Lett.

[91] Chan SY, Zhang YY, Hemann C, Mahoney CE, Zweier JL, Loscalzo J (2009). MicroRNA-210 controls mitochondrial metabolism during hypoxia by repressing the iron-sulfur cluster assembly proteins ISCU1/2. Cell Metab.

[92] Kuan CY, Chen HR, Gao N (2021). Brain-targeted hypoxia-inducible factor stabilization reduces neonatal hypoxic-ischemic brain injury. Neurobiol Dis.

[93] Semenza GL (2013). HIF-1 mediates metabolic responses to intratumoral hypoxia and oncogenic mutations. J Clin Invest.

[94] Barteczek P, Li L, Ernst AS, Böhler LI, Marti HH, Kunze R (2017). Neuronal HIF-1α and HIF-2α deficiency improves neuronal survival and sensorimotor function in the early acute phase after ischemic stroke. J Cereb Blood Flow Metab.

[95] Terpolilli NA, Feiler S, Dienel A (2016). Nitric oxide inhalation reduces brain damage, prevents mortality, and improves neurological outcome after subarachnoid hemorrhage by resolving early pial microvasospasms. J Cereb Blood Flow Metab.

[96] Zhang J, Han Y, Wang Y, Cheng X, Wang CJ (2018). Neuronal nitric oxide synthase inhibition reduces brain damage by promoting collateral recruitment in a cerebral hypoxia-ischemia mice model. Eur Rev Med Pharmacol Sci.

[97] Gainutdinov KL, Kulchitsky VA, Andrianov VV (2022). Application of EPR spectroscopy to study the content of NO and copper in the frontal lobes, hippocampus, and liver of rats after cerebral ischemia. Tech Phys.

[98] Gainutdinov KL, Andrianov VV, Yafarova GG (2024). Content of nitrogen monoxide and copper in the hippocampus of a rat model of short-term cerebral ischemia followed by reperfusion. Biophysics.

[99] Zhang RL, Zhang ZG, Chopp M (2013). Targeting nitric oxide in the subacute restorative treatment of ischemic stroke. Expert Opin Investig Drugs.

[100] Soloveva AG, Peretyagin PV (2022). The state of nitrogen metabolism under the influence of exogenous NO in experimental thermal trauma. Opera Med Physiol.

[101] Huang R, Zhang C, Wang X, Hu H (2021). PPARγ in ischemia-reperfusion injury: overview of the biology and therapy. Front Pharmacol.

[102] Sukmansky OI, Reutov VP (2016). Gasotransmitters: physiological role and involvement in the pathogenesis of the diseases. Usp Fiziol Nauk.

[103] Kader A, Frazzini VI, Solomon RA, Trifiletti RR (1993). Nitric oxide production during focal cerebral ischemia in rats. Stroke.

[104] Mikoyan VD, Kubrina LN, Serezhenkov VA, Stukan RA, Vanin AF (1997). Complexes of Fe2+ with diethyldithiocarbamate or N-methyl-d-glucamine dithiocarbamate as traps of nitric oxide in animal tissues: comparative investigations. Biochim Biophys Acta Gen Subj.

[105] Sato S, Tominaga T, Ohnishi T, Ohnishi ST (1994). Electron paramagnetic resonance study on nitric oxide production during brain focal ischemia and reperfusion in the rat. Brain Res.

[106] Iadecola C, Zhang F, Casey R, Nagayama M, Ross ME (1997). Delayed reduction of ischemic brain injury and neurological deficits in mice lacking the inducible nitric oxide synthase gene. J Neurosci.

[107] Yafarova G, Tokalchik Y, Filipovich T (2023). The effects of intranasal implantation of mesenchymal stem cells on nitric monoxide levels in the hippocampus, control of cognitive functions, and motor activity in a model of cerebral ischemia in rats. BioNanoSci.

[108] Andrianov VV, Kulchitsky VA, Yafarova GG (2023). Investigation of NO role in neural tissue in brain and spinal cord injury. Molecules.

[109] Willmot M, Gray L, Gibson C, Murphy S, Bath PM (2005). A systematic review of nitric oxide donors and L-arginine in experimental stroke; effects on infarct size and cerebral blood flow. Nitric Oxide.

[110] Chen ZQ, Mou RT, Feng DX, Wang Z, Chen G (2017). The role of nitric oxide in stroke. Med Gas Res.

[111] Vanin AF (2023). Dinityrosyl iron complexes with thiol-containing ligands as a functionally active “working form” of nitric oxide system in living organisms: a review. Mol Biol (Mosk).

[112] Andrianov VV, Schepetkin IA, Bazan LV (2024). Evaluation of nitric oxide-donating properties of 11h-indeno[1,2-b]quinoxalin-11-one oxime (IQ-1) by electron paramagnetic resonance spectroscopy. Molecules.

[113] Samdani AF, Dawson TM, Dawson VL (1997). Nitric oxide synthase in models of focal ischemia. Stroke.

[114] Eliasson MJ, Huang Z, Ferrante RJ (1999). Neuronal nitric oxide synthase activation and peroxynitrite formation in ischemic stroke linked to neural damage. J Neurosci.

[115] Chen Y, Irie Y, Keung WM, Maret W (2002). S-nitrosothiols react preferentially with zinc thiolate clusters of metallothionein III through transnitrosation. Biochemistry.

[116] Dawson DA, Kusumoto K, Graham DI, McCulloch J, Macrae IM (1992). Inhibition of nitric oxide synthesis does not reduce infarct volume in a rat model of focal cerebral ischaemia. Neurosci Lett.

[117] Sancesario G, Iannone M, Morello M, Nisticò G, Bernardi G (1994). Nitric oxide inhibition aggravates ischemic damage of hippocampal but not of NADPH neurons in gerbils. Stroke.

[118] Yamamoto S, Golanov EV, Berger SB, Reis DJ (1992). Inhibition of nitric oxide synthesis increases focal ischemic infarction in rat. J Cereb Blood Flow Metab.

[119] Jung KH, Chu K, Ko SY (2006). Early intravenous infusion of sodium nitrite protects brain against in vivo ischemia-reperfusion injury. Stroke.

[120] Evgenov OV, Pacher P, Schmidt PM, Haskó G, Schmidt HH, Stasch JP (2006). NO-independent stimulators and activators of soluble guanylate cyclase: discovery and therapeutic potential. Nat Rev Drug Discov.

[121] Godínez-Rubí M, Rojas-Mayorquín AE, Ortuño-Sahagún D (2013). Nitric oxide donors as neuroprotective agents after an ischemic stroke-related inflammatory reaction. Oxid Med Cell Longev.

[122] Wang YZ, Li TT, Cao HL, Yang WC (2019). Recent advances in the neuroprotective effects of medical gases. Med Gas Res.

[123] Andrianov VV, Pashkevich SG, Yafarova GG (2016). Changes of nitric oxide content in the rat hippocampus, heart and liver in acute phase of ischemia. Appl Magn Reson.

[124] Calabrese V, Mancuso C, Calvani M, Rizzarelli E, Butterfield DA, Stella AM (2007). Nitric oxide in the central nervous system: neuroprotection versus neurotoxicity. Nat Rev Neurosci.

[125] Granger DN, Kvietys PR (2015). Reperfusion injury and reactive oxygen species: the evolution of a concept. Redox Biol.

[126] Chan KL (2002). Role of nitric oxide in ischemia and reperfusion injury. Curr Med Chem Anti Inflamm Anti Allergy Agents.

[127] Luo Y, Duan X, Bian L, Chen Z, Kuang L, Li Y (2019). Ischemic preconditioning preventing downregulation of miR-182 protects intestine against ischemia/reperfusion injury by inhibiting apoptosis. Arch Med Res.

[128] Yan HF, Tuo QZ, Yin QZ, Lei P (2020). The pathological role of ferroptosis in ischemia/reperfusion-related injury. Zool Res.

[129] Soares ROS, Losada DM, Jordani MC, Évora P, Castro ESO (2019). Ischemia/reperfusion injury revisited: an overview of the latest pharmacological strategies. Int J Mol Sci.

[130] Andrianov VV, Yafarova GG, Pashkevich SG (2020). Changes of the nitric oxide and copper content in the olfactory bulbs of rat brain after modeling of brain stroke and intranasal administration of mesenchymal stem cells. Appl Magn Reson.

[131] Truzzi DR, Medeiros NM, Augusto O, Ford PC (2021). Dinitrosyl iron complexes (DNICs). From spontaneous assembly to biological roles. Inorg Chem.

[132] Moroz LL, Kohn AB (2011). Parallel evolution of nitric oxide signaling: diversity of synthesis and memory pathways. Front Biosci (Landmark Ed).

[133] Kumar S, Singh RK, Bhardwaj TR (2017). Therapeutic role of nitric oxide as emerging molecule. Biomed Pharmacother.

[134] Lehnert N, Kim E, Dong HT (2021). The biologically relevant coordination chemistry of iron and nitric oxide: electronic structure and reactivity. Chem Rev.

[135] Shumaev KB, Gubkin AA, Serezhenkov VA (2008). Interaction of reactive oxygen and nitrogen species with albumin- and methemoglobin-bound dinitrosyl-iron complexes. Nitric Oxide.

[136] Abat JK, Saigal P, Deswal R (2008). S-Nitrosylation - another biological switch like phosphorylation?. Physiol Mol Biol Plants.

[137] Truzzi DR, Alves SV, Netto LES, Augusto O (2020). The peroxidatic thiol of peroxiredoxin 1 is nitrosated by nitrosoglutathione but coordinates to the dinitrosyl iron complex of glutathione. Antioxidants (Basel).

[138] Shumaev KB, Kosmachevskaya OV, Grachev DI (2021). Possible mechanism of antioxidant action of dinitrosyl iron complexes. Biomed Khim.

[139] Mukosera GT, Clark TC, Ngo L (2020). Nitric oxide metabolism in the human placenta during aberrant maternal inflammation. J Physiol.

[140] Vanin AF (2025). Principal role of EPR method in discovery, identification and study of dinitrosyl iron complexes in living organisms (review). Appl Magn Reson.

[141] Vanin AF, Tkachev NA, Mikoyan VD, Raevskaya VE, Asanbaeva NB, Bagryanskaya EG (2025). The nature of thiol-containing ligands in dinitrosyl iron complexes as a factor determining the stability of these complexes. Biophysics.

[142] Chazov EI, Rodnenkov OV, Zorin AV (2012). Hypotensive effect of Oxacom® containing a dinitrosyl iron complex with glutathione: animal studies and clinical trials on healthy volunteers. Nitric Oxide.

[143] Vanin AF, Abramov AA, Timoshin AA, Lakomkin VL, Tkachev NA (2024). Stability of hypotensive activity in freeze-dried preparations of dinitrozyl iron complexes with glutathione (“Oxacom”) throughout the period of storage for 15 years at ambient temperature. Biophysics.

[144] Vanin AF, Ostrovskaya LA, Korman DB (2025). Binuclear dinitrosyl iron complexes with cysteine inhibite the development of experimental tumor. Biophysics.

[145] Igrunkova A, Fayzullin A, Serejnikova N (2023). Beneficial effects of dinitrosyl iron complexes on wound healing compared to commercial nitric oxide plasma generator. Int J Mol Sci.

[146] Remizova MI, Kochetygov NI, Gerbout KA (2011). Effect of dinitrosyl iron complexes with glutathione on hemorrhagic shock followed by saline treatment. Eur J Pharmacol.

[147] Gainutdinov KL, Andrianov VV, Iyudin VS (2013). EPR study of nitric oxide production in rat tissues under hypokinesia. Biophysics.

[148] Vanin AF (2020). How is nitric oxide (NO) converted into nitrosonium cations (NO(+)) in living organisms? (Based on the results of optical and EPR analyses of dinitrosyl iron complexes with thiol-containing ligands). Appl Magn Reson.

[149] Vanin AF (2020). Dinitrosyl iron complexes with thiol-containing ligands can suppress viral infections as donors of the nitrosonium cation (hypothesis). Biophysics (Oxf).

[150] Vanin AF, Tronov VA, Trifonova NE, Nekrasova EI, Mikoyan VD, Tkachev NA (2025). Nitrosonium cation as a nitroprusside constituent determining its cytotoxicity. Biophysics.

[151] Titov VY, Dolgorukova AM, Osipov AN, Kochish, II (2023). DNIC is a structure providing specificity of physiological effects of NO. Bull Exp Biol Med.

[152] Gainutdinov KL, Pashkevich SG, Andrianov VV (2017). Participation of NO-synthase in Control of Nitric Oxide Level in Rat Hippocampus after Modelling of Ischaemic and Haemorrhagic Insult. BioDiscovery.

[153] Deryagin OG, Gavrilova SA, Buravkov SV (2018). The role of ATP-sensitive potassium channels and nitric oxide in the protective effect of preconditioning of the brain. Neurosci Behav Physiol.

[154] Gertz K, Endres M (2008). ENOS and stroke: prevention, treatment and recovery. Future Neurol.

